# Evaluation of MycAssay™ Aspergillus for Diagnosis of Invasive Pulmonary Aspergillosis in Patients without Hematological Cancer

**DOI:** 10.1371/journal.pone.0061545

**Published:** 2013-04-19

**Authors:** Jesús Guinea, Camilo Padilla, Pilar Escribano, Patricia Muñoz, Belén Padilla, Paloma Gijón, Emilio Bouza

**Affiliations:** 1 Clinical Microbiology and Infectious Diseases Department, Hospital General Universitario Gregorio Marañón, Universidad Complutense de Madrid, Madrid, Spain; 2 Instituto de Investigación Sanitaria del Hospital Gregorio Marañón, Madrid, Spain; 3 CIBER de Enfermedades Respiratorias (CIBERES CD06/06/0058), Palma de Mallorca, Spain; 4 Medicine Department, School of Medicine, Universidad Complutense de Madrid, Madrid, Spain; University of Wisconsin Medical School, United States of America

## Abstract

Methods based on real-time polymerase chain reaction (PCR) can speed up the diagnosis of invasive aspergillosis but are limited by a lack of standardization. We evaluated the commercially available MycAssay™ Aspergillus test for the diagnosis of invasive aspergillosis in patients without hematological cancer. We prospectively collected 322 lower respiratory tract samples (November 2009–January 2011) from 175 patients with lower respiratory tract infection and the following predisposing conditions: solid cancer (16.8%), cirrhosis (16.8%), corticosteroid therapy (71.7%), HIV infection (15.6%), chronic obstructive pulmonary disease (COPD, 52.6%), solid organ transplantation (kidney [1.2%], heart [3%], liver [4.6%]), or none (3.5%). Specimens were obtained when clinically indicated and analyzed in the microbiology laboratory. *Aspergillus* DNA was extracted and amplified by means of MycXtra® and MycAssay™ Aspergillus. *Aspergillus* spp. was isolated from 65 samples (31 patients). According to the European Organization for Research and Treatment of Cancer and Bulpa's criteria (for patients with COPD), 15 had probable invasive aspergillosis. MycAssay™ Aspergillus results were negative (n = 254), positive (n = 54), or indeterminate (n = 14). The sensitivity, specificity, positive predictive value, negative predictive value, and diagnostic odds ratio of the MycAssay™ (first sample/any sample) were 86.7/93, 87.6/82.4, 34.1/34.1, 92.2/100, and 48/68.75. The differences between the proportion of samples with positive PCR determinations (63%) and the proportion of samples with *Aspergillus* spp. isolation (75%) did not reach statistical significance (*P* = 0.112). The median time from sample culture to visualization of fungal growth was 3 days, compared with ∼4 hours for MycAssay™ Aspergillus PCR. MycAssay™ Aspergillus showed high sensitivity for the diagnosis of invasive aspergillosis in patients without hematological cancer. Sensitivity increased when multiple samples were used. Compared with fungal culture, PCR significantly reduced the time to diagnosis.

## Introduction

Invasive aspergillosis is an opportunistic infection affecting patients with various degrees of immunosuppression. Patients with deep and prolonged neutropenia have traditionally had the highest risk of invasive aspergillosis [1], [2], [3], [4], [5]. Other at-risk groups include solid organ transplant recipients, patients with chronic obstructive pulmonary disease (COPD), patients receiving corticosteroids or other immunosuppressive agents, and patients with liver cirrhosis [6], [7].

The number of patients without hematological malignancies who are affected by invasive aspergillosis is increasing. Mortality in this group is high, probably because of the low index of suspicion of the infection and the consequent delay in diagnosis [8], [9], [10], [11]. As a favorable outcome depends on prompt and appropriate antifungal therapy, rapid diagnosis is increasingly important.

Detection of galactomannan in serum has proven useful for the diagnosis of invasive aspergillosis in neutropenic patients; unfortunately, its sensitivity is below 50% in patients without neutropenia [12], [13], [14], [15], [16]. Detection of galactomannan in bronchoalveolar lavage (BAL) samples is more sensitive than detection in serum samples, although BAL samples are not always available [15]. The isolation of *Aspergillus* in lower respiratory tract samples from non-neutropenic patients is often the first microbiological evidence of invasive pulmonary aspergillosis. However, as culture is slow, detection of *Aspergillus* in clinical samples is delayed.

Methods based on real-time polymerase chain reaction (PCR) can speed up the diagnosis of invasive aspergillosis but are limited by a lack of standardization [17], [18]. MycAssay™ Aspergillus is a recently marketed real-time PCR technique for detection of *Aspergillus* DNA in lower respiratory tract samples. This assay has been studied mostly in BAL samples from patients with hematological malignancies or those admitted to intensive care units [19].

In the present study, we evaluated the MycAssay™ Aspergillus test in respiratory samples, including BAL, spontaneous sputum, and bronchial aspirate, for the diagnosis of invasive aspergillosis in patients without hematological cancer.

## Materials and Methods

### Patients and clinical samples

From November 2009 to January 2011, we recruited 175 patients with one or more lower respiratory samples submitted to the microbiology laboratory. Most of the patients (96.5%) had clinical suspicion of lower respiratory tract infection and at least one invasive pulmonary aspergillosis host factor, excluding hematological cancer. A total of 322 samples were collected. Samples with indeterminate results were retested, and the second result was chosen. Samples showing a confirmatory indeterminate PCR result were excluded from the analysis (n = 14; 4.3%). The number of samples studied/collected was as follows: spontaneous sputum (n = 142/145), bronchial aspirate (n = 104/111), BAL (n = 61/65), and protected brush catheter (n = 1/1).

Two patients had a single sample, each with an indeterminate result, and were excluded from the analysis. The remaining 173 patients were classified as having or not having invasive pulmonary aspergillosis or other mold infection according to the revised criteria of the European Organization for Research and Treatment of Cancer (EORTC) [20], [21] or Bulpa's criteria (exclusively for patients with COPD) [20], [21]. Colonization was defined as the isolation of *Aspergillus* spp. in lower respiratory samples in patients not meeting the EORTC or Bulpa's criteria. Cirrhosis was included as a host factor, since invasive aspergillosis has been found in critically ill patients with cirrhosis and no other predisposing conditions [8]. The predisposing conditions for invasive aspergillosis were active solid cancer (16.8%), cirrhosis (16.8%), corticosteroid consumption (71.7%), HIV infection (15.6%), COPD (52.6%), solid organ transplantation (kidney [1.2%], heart [3%], liver [4.6%]), neutropenia, (4.6%), or none (3.5%). A high proportion of the patients (90%) were consuming antibiotics when the sample was collected.

All samples were obtained only when clinically indicated, and no additional samples were requested for the study. The samples were prospectively collected and the patients' charts were retrospectively reviewed. Clinicians were blinded to the PCR result, which was not included as a microbiological diagnostic criterion.

### Sample processing, genomic *Aspergillus* DNA extraction, and amplification using MycAssay™ Aspergillus

Samples were split for fungal culture and DNA extraction. All specimens were processed for *Aspergillus* DNA detection, and most (n = 298/308; 97%) were cultured on both bacteria and fungal media. BAL samples (1 mL) were centrifuged at 3,000 *g* for 10 minutes. The concentrated pellets obtained were processed for fungal culture and streaked on culture media plates. Sputum and bronchial aspirate were converted to fluid by the addition of acetylcysteine (Pharmazam, Spain) and added to the agar plates using a sterile loop (10 µL). The fungal culture media were Sabouraud-dextrose agar with chloramphenicol and brain-heart infusion agar with antibacterial agents. The bacterial culture media were sheep blood agar and chocolate agar. Filamentous fungal isolates were identified according to standard morphological procedures.

Samples for DNA extraction were stored frozen at −20°C until batch analysis. Thick mucous samples, such as sputum and bronchial aspirate, were converted to fluid by the addition of BBL™ MycoPrep™ Reagent (Becton Dickinson, Shannon, Ireland). These samples and BAL samples (1 mL) were then centrifuged at 3,000 *g* for 20 minutes. The pellets obtained were processed for *Aspergillus* DNA extraction using the manual MycXtra® fungal DNA extraction kit (Myconostica, now a Lab21 company, Cambridge, UK), which includes a mechanical disruption step. BAL supernatants were stored for galactomannan determination.

Purified extracted genomic *Aspergillus* DNA was further amplified using the MycAssay™ Aspergillus kit (Myconostica, now a Lab21 company, Cambridge, UK) in the Cepheid SmartCycler® platform (Cepheid, Sunnyvale, California, USA). MycAssay™ Aspergillus was designed for the detection of genomic DNA from 18 different *Aspergillus* species (including *A. fumigatus*, *A. flavus*, *A. terreus*, and *A. niger*) using molecular beacons. The assay targets the 18S rRNA gene and contains an internal control of plant origin to avoid false-negative results due to the presence of PCR inhibitors.

Briefly, 10 µL of the extracted DNA was mixed with the amplification reagents in a final reaction volume of 25 µL. PCR results for each sample were reported as negative (samples without *Aspergillus* DNA amplification with positive amplification of the internal control), positive (samples with *Aspergillus* DNA amplification), or indeterminate (samples with negative *Aspergillus* DNA amplification and failure of amplification of the internal control). The MycXtra® DNA extraction kit and MycAssay™ Aspergillus were applied according to the manufacturer's instructions [22]. The crossing point (Cp) was the cycle number at which the real-time PCR test became positive.

### Data analysis

None of the patients had proven invasive pulmonary aspergillosis; only probable invasive aspergillosis was considered to be a true infection. We calculated the sensitivity, specificity, positive predictive value (PPV), negative predictive value (NPV), likelihood ratio for a positive result (LR+), likelihood ratio for a negative result (LR−), and diagnostic odds ratio (DOR) of the PCR for the diagnosis of invasive pulmonary aspergillosis. As multiple respiratory samples were studied in several patients, the diagnostic values of the PCR were calculated based both on the results of the first sample submitted to the microbiology laboratory and on the results of any sample from the same patient. PCR and fungal culture were described and compared using the chi-square test and a standard binomial method for calculation of 95% confidence intervals.

### Ethics statement

This study was approved by the local ethics committee (Comité Ético de Investigación Clínica (CEIC-A1)). The participants provided their written informed consent to participate in the study. All patient data were anonymized after collection.

## Results

The 173 patients studied were mostly male (76.3%) and had a mean age of 60.8±19.5 (2–96) years. Patients were classified as having probable invasive aspergillosis (n = 15; 8.7%), possible invasive aspergillosis (n = 3; 1.7%), *Aspergillus* colonization (clinically non-significant *Aspergillus* isolation [n = 21; 12.2%], pulmonary scedosporiosis [n = 1; 0.6%]), or non-invasive mold infection. The 15 patients with probable invasive aspergillosis are summarized in Table 1. At the time of sample collection, all patients had fever that did not respond to broad-spectrum antibiotics, and 80% required admission to the intensive care unit. A high proportion (40%) had COPD as the underlying predisposing condition. In 14 out of the 15 cases, the lung was the only organ infected. Serum galactomannan determination was only positive in 5 (35.7%) out of the 14 patients in whom it was applied.

**Table 1:** Patients with probable invasive aspergillosis: underlying conditions, organs affected, and microbiological findings.

Patient	Admission to ICU^a^	Sample Obtained in the ICU	Main predisposing condition	Organ affected	TAC findings	Serum GM>0.5^c^	BAL GM	MycAssay result on first sample	MycAssay result on any sample	*Aspergillus* species isolated in any of the clinical samples	Clinical outcome	Antifungal treatment
1	**Yes**	**Yes**	COPD^b^	Lung	ND	Negative	No BAL sample	**Positive**	**Positive**	*A. terreus*	Poor	Voriconazole
2	**Yes**	**Yes**	COPD	Lung	Bilateral and multifocal nodules	Negative	No BAL sample	**Positive**	**Positive**	*A. flavus*	Poor	Voriconazole+caspofungin
3	**Yes**	**Yes**	Solid tumor	Lung	Bilateral and multifocal nodular consolidation	**Positive**	No BAL sample	**Positive**	**Positive**	*A. fumigatus*	Poor	Voriconazole
4	**Yes**	**Yes**	HIV	Lung	ND	**Positive**	No BAL sample	**Positive**	**Positive**	*A. nidulans*+*A. niger*	Poor	Voriconazole+caspofungin
5	No	**Yes**	Tracheal prosthesis	Trachea	ND	Negative	No BAL sample	**Positive**	**Positive**	*A. niger+A. terreus*	Favorable	Voriconazole
6	**Yes**	No	COPD	Lung	ND	Negative	No BAL sample	**Positive**	**Positive**	*A. fumigatus+A. terreus*	Poor	Caspofungin
7	No	No	Solid tumor	Lung	ND	Negative	No BAL sample	**Positive**	**Positive**	*A. fumigatus*	Poor	Voriconazole
8	**Yes**	**Yes**	COPD	Lung	ND	Negative	**4 ng/ml**	**Positive**	**Positive**	*A. fumigatus*	Favorable	Voriconazole
9	No	No	Corticosteroids	Lung	Bilateral diffuse infiltrates	Negative	No BAL sample	**Positive**	**Positive**	*A. fumigatus*	Poor	Voriconazole
10	**Yes**	**Yes**	Corticosteroids+Liver cirrhosis	Lung	ND	**Positive**	No BAL sample	**Positive**	**Positive**	*A. niger*	Poor	Voriconazole+amphotericin B
11	**Yes**	**Yes**	Corticosteroids+Liver cirrhosis	Lung	ND	**Positive**	**4 ng/ml**	**Positive**	**Positive**	*A. fumigatus*	Poor	Voriconazole
12	**Yes**	No	Corticosteroids+Liver cirrhosis	Lung	ND	ND^d^	No BAL sample	**Positive**	**Positive**	*A. fumigatus*	Poor	Caspofungin
13	**Yes**	**Yes**	COPD+solid tumor	Lung	Multiple lung infiltrates and cavitations	**Positive**	**2.765**	**Positive**	**Positive**	*A. fumigatus*	Poor	Voriconazole
14	**Yes**	**Yes**	Corticosteroids	Lung	Bilateral lung infiltrates	Negative	**0.63**	Negative	Negative	*A. fumigatus*	Poor	Voriconazole
15	**Yes**	**Yes**	HIV	Lung	Bilateral lung infiltrates	Negative	No BAL sample	Negative	**Positive**	*A. fumigatus*	Favorable	Voriconazole+amphotericin B

*Aspergillus* spp. were isolated in 63/308 (22%) samples from 31 patients. The species distribution was as follows: *A. fumigatus* (n = 45), *A. niger* (n = 10), *A. terreus* (n = 7), *A. flavus* (n = 5), and other (n = 3) spp. *Aspergillus* spp. was isolated in one or more clinical samples from the 15 patients with probable invasive aspergillosis.

MycAssay™ Aspergillus was positive in 54/254 (17.5%) samples, and in at least one sample from 14 of the 15 patients with invasive aspergillosis. Cp values of positive PCR determinations were lower in samples from patients with probable aspergillosis than in samples from patients with clinically non-significant *Aspergillus* (30.18±3.3 vs. 33±2.66; *P* = 0.001). This finding indicated a higher load of *Aspergillus* spp. in patients with invasive aspergillosis than in those without.

As expected, the proportion of samples with *Aspergillus* isolation or with positive PCR results was higher in patients with invasive aspergillosis (Table 2). Concordance between fungal culture and PCR was high in all samples and in samples from patients with or without invasive aspergillosis (83.9%, 86.5%, and 83.5%, respectively). Discrepancies were mostly found in samples yielding *Aspergillus* without DNA amplification (approximately 10% of samples). Interestingly, most discrepancies were found in samples from patients without invasive aspergillosis (PCR-negative/culture-positive results, 86%; PCR-positive/culture-negative results, 94.4%). The samples with PCR-positive/culture-negative results showed higher Cp values than the samples in which culture and PCR were concordant. The analysis of samples from patients with invasive aspergillosis indicated that the proportion of samples in which the PCR result was positive (63%) did not differ from the proportion of samples in which *Aspergillus* spp. was isolated (75%) (*P* = 0.615). The results of the comparison of PCR and fungal culture with BAL, sputum, and bronchial aspirate samples are shown in Table 3. The performance of PCR and fungal culture did not differ with the type of sample studied (*P*>0.05).

**Table 2:** Comparison of fungal culture and MycAssay™ Aspergillus for all samples and for samples from patients with or without invasive aspergillosis.

	All patients (n = 173)	Patients with IPA^a^ (n = 15)	Patients without IPA (n = 158)
**Cultured samples**	298^b^	37	261
Positive (% and 95% CI)	63 (21.1% 16–26%)	27 (73%, 58–88%)	36 (13.8%, 10–18%)
**Samples with MycAssay determinations**	308	37	271
Positive (% and 95% CI)^c^	51(16.5%, 13–21%)	24 (64.9%; 49–81%)	27 (10.3%, 7–14%)
**Concordance between fungal**			
**culture and MycAssay** d			
Culture −/MycAssay − (% and 95% CI)	217 (72.8%, 68–78%)	9 (24.3%, 10–39%)	208 (79.7%, 75–85%)
Culture +/MycAssay + (% and 95% CI)	33 (11.1%, 7–15%)	23 (62.2%, 46–79%)	10 (3.8%, 1–6%)
Culture −/MycAssay + (% and 95% CI)	18 (6%, 3–9%)	1 (2.7%, −3–8%)	17 (6.5%, 3–10%)
Culture +/MycAssay − (% and 95% CI)	30 (10.1%, 7–14%)	4 (10.8%, 0–21%)	26 (10%, 6–14%)

The table shows the proportion of samples with positive results and the concordance between both procedures for all samples and for samples from patients with and without invasive aspergillosis.

a IPA, invasive pulmonary aspergillosis.

b Fungal or bacterial culture was performed in 298 out of the 308 samples studied.

c CI, confidence interval.

d The analysis of concordance was performed in 298 samples after excluding the remaining 10 samples in which only PCR was performed.

**Table 3:** Comparison between fungal culture and MycAssay™ Aspergillus according to the type of lower respiratory tract sample studied.

	Bronchoalveolar lavage	Sputum	Bronchial aspirate
**Cultured samples** a	61	136	101
Positive (% and 95% CI)^b^	8 (13.1%, 4–22%)	36 (26.5%, 19–34%)	19 (18.8%, 11–27%)
**Samples with MycAssay determinations**	61	143	104
Positive (% and 95% CI)	7 (11.5%, 3–20%)	27 (18.9%, 11.24%)	20 (19.2%, 12–28%)
**Concordance between fungal**			
**culture and MycAssay** c			
Culture −/MycAssay − (% and 95% CI)	49 (80.3%, 70–91%)	90 (66.2%, 58–74%)	78 (77.2%, 69–86%)
Culture +/MycAssay + (% and 95% CI)	3 (4.9%, −1–11%)	14 (10.3%, 5–15%)	16 (15.8%, 9–23%)
Culture −/MycAssay + (% and 95% CI)	4 (6.6%, 0–13%)	10 (7.4%, 3–12%)	4 (4%, 0–8%)
Culture +/MycAssay − (% and 95% CI)	5 (8.2%, 1–15%)	22 (16.2%, 10–22%)	3 (3%, 0–6%)

The table shows the proportions of samples with PCR− or fungal culture–positive results and the concordance between both procedures for bronchoalveolar lavage samples, sputum samples, and bronchial aspirate samples.

a Fungal or bacterial culture was performed in 298 of the 308 samples studied.

b CI, confidence interval

c The analysis of concordance was performed in 298 samples after excluding the remaining 10 samples in which only PCR was performed.

The sensitivity, specificity, PPV, NPV, LR+, LR−, and DOR of the MycAssay™ Aspergillus and fungal culture for the diagnosis of invasive pulmonary aspergillosis performed on lower respiratory samples of patients are shown in Tables 4 and 5. Sensitivity was higher when multiple samples per patient were studied; however, analysis of multiple samples did not affect specificity to any extent. In order to study how the diagnostic value of the PCR could be affected in different situations, patients were divided into the following groups: patients with COPD (n = 91), patients with infection not improving with antibiotics (n = 28), and patients in the intensive care unit with pneumonia (n = 35). Sensitivity and specificity remained unaffected by this stratification. MycAssay™ Aspergillus results were available approximately 4 hours after sample reception. In contrast, the number of days to visualization of fungal growth in the microbiological culture plates was as follows: mean, n = 4.3±4.1; median, n = 3; and mode, n = 2.

**Table 4:** Sensitivity, specificity, LR+, LR−, and DOR of MycAssay™ Aspergillus for the diagnosis of invasive pulmonary aspergillosis performed on lower respiratory samples from patients without hematological cancer.

	First sample		Any sample	
	Fungal culture	MycAssay™	Fungal culture	MycAssay™
**All patients (n = 173)**				
Sensitivity	86.7	86.7	100	93.3
Specificity	83.5	88	81.6	82.9
LR+	5.3	7.2	5.4	5.5
LR−	0.16	0.15	0	0.08
DOR	33.13	48	∞	68.75
**COPD patients (n = 91)**				
Sensitivity	100	100	100	100
Specificity	77.6	85.9	74.1	78.8
LR+	4.5	6.2	3.9	4.7
LR−	0	0	0	0
DOR	∞	∞	∞	∞

A sub-analysis including only patients with chronic obstructive pulmonary disease is shown.

LR+, likelihood ratio for a positive result; LR−, likelihood ratio for a negative result; DOR, diagnostic odds ratio; COPD, chronic obstructive pulmonary disease.

**Table 5:** Positive predictive value (PPV) and negative predictive value (NPV) of the MycAssay™ Aspergillus and *Aspergillus* culture for the diagnosis of invasive aspergillosis in different clinical situations.

	No. of patients	No. of patients with invasive aspergillosis (prevalence)	MycAssay™ Aspergillus/*Aspergillus* culture	
			PPV	NPV
**All patients**	173	15 (8.7%)	34.1/34.1	99.2/100
**Patients with COPD**	91	6 (6.6%)	25.0/21.4	100/100
**Patients in ICU with pneumonia**	35	12 (34%)	78.6/80	95.2/100
**Patients with infection not improving with antibiotics**	28	15 (53.6%)	87.5/83.3	91.6/100

The analysis was performed based on the results of any of the studied samples.

In 14 out of the 15 patients with probable invasive pulmonary aspergillosis, the samples were taken before the initiation of antifungal treatment, and no additional samples were studied during antifungal treatment. Serial samples from the remaining patient (no. 15, Table 1) were studied; of the five samples taken during antifungal treatment, three were positive for MycAssay and two were negative. Interestingly, after the samples became negative for MycAssay, the patient's clinical condition improved. Of the three patients with possible invasive aspergillosis, one received voriconazole and improved, and two did not receive antifungal treatment and died. The result of MycAssay™ Aspergillus was negative for the samples from the three patients with possible invasive aspergillosis.

## Discussion

The spectrum of patients at risk of invasive pulmonary aspergillosis has expanded in recent years because of an increase in the number of patients with predisposing conditions other than hematological disorders. This expansion has been illustrated in studies conducted at large tertiary hospitals where case collection was not restricted to the hematology ward and intensive care units and where a large number of autopsies are performed [6], [8], [23].

Invasive aspergillosis in patients with non-hematological malignancies is characterized by high mortality (60–100%) [6], [10], [11], which probably reflects the limitations of current diagnostic tools based on radiology or microbiology and the low index of clinical suspicion.

Diagnosis of invasive aspergillosis is based on a combination of compatible clinical findings in patients with risk factors, together with histopathological evidence of invasion, radiological findings, isolation of *Aspergillus* spp in lower respiratory tract samples, or detection of circulating biomarkers in fluids. Histopathology is necessary to obtain a definitive diagnosis, although lung biopsies are rarely obtained [24]. Compared with culture and molecular diagnostic testing, the accuracy of histomorphological diagnosis is at best 80% [25]. The radiological findings that are common in patients with neutropenia and invasive aspergillosis are infrequent in non-neutropenic patients [8], [26]. Culture of lower respiratory tract samples from patients with clinical suspicion is still widely used, although it is slow and limited by low sensitivity and specificity [27]. Furthermore, the detection of circulating galactomannan in serum samples from non-neutropenic patients has a low sensitivity for the diagnosis of invasive aspergillosis [6], [14], [16].

In this scenario, the development of fast, sensitive, and specific diagnostic procedures to detect *Aspergillus* in respiratory tract samples from patients without hematological malignancies is attractive. One of the most encouraging new procedures is DNA *Aspergillus* detection by real-time PCR assays. In our study, PCR results were available as quickly as 4 hours after sample collection, which is a must faster turnaround than the 3 days required to observe growth of *Aspergillus* in culture. However, the time from sample culture and detection of fungal growth could be reduced by inspecting plates every day, even at the weekend. Furthermore, simultaneous amplification of DNA on respiratory samples and fungal culture will allow us to identify isolates to species level and perform antifungal susceptibility testing on isolates.

Detection of *Aspergillus* DNA in BAL samples is encouraging and confirms a diagnosis of invasive aspergillosis in more patients than conventional procedures [28]. It has been studied mostly in patients with hematological disorders [29], [30], [31]. A recent meta-analysis showed a sensitivity and specificity of 0.91 and 0.92, respectively, but highlighted the lack of standardization [32]. Nevertheless, the role of PCR in respiratory tract samples from non-hematological patients requires further evaluation. BAL samples were collected in 4 out of the 15 patients with probable invasive aspergillosis. In all cases, the BAL galactomannan concentrations were above 0.5 ng/ml.

The lack of standardization of the *Aspergillus* PCR to date has hampered its introduction in the defining criteria for probable aspergillosis [20]. MycAssay™ Aspergillus is a commercially available real-time PCR for the detection of the most clinically relevant *Aspergillus* species [22]. As a fully standardized CE-marked test with full manufacturing quality controls, MycAssay™ Aspergillus fulfils the requirements for standardization of *Aspergillus* PCR necessary to confirm a diagnosis of invasive aspergillosis. MycAssay™ Aspergillus was previously assayed on BAL samples [19], sputum samples [33], [34], and tissue samples [35]. MycAssay can be performed in the clinical microbiology laboratory by personnel trained in molecular biology technology; the estimated cost of the test per determination in Spain is €25–30.

We evaluated MycAssay™ Aspergillus on lower respiratory tract samples (including BAL, spontaneous sputum, and bronchial aspirate) from patients with predisposing conditions other than hematological malignancy and clinical suspicion of invasive aspergillosis. We found that the three kinds of lower respiratory tract samples studied were suitable for detection of *Aspergillus* spp. DNA. Our gold standard was clinical in that we classified patients using the criteria proposed by the EORTC [20], [21] or by Bulpa [20], [21]. The sensitivity of the PCR was high and increased when several samples per patient were studied; specificity was not affected by including multiple samples per patient. Sensitivity was also high in patients with COPD, a predisposing condition in a large proportion of the patients studied. MycAssay™ Aspergillus showed a high NPV, thus suggesting that a PCR-negative result in any lower respiratory tract sample from a patient without hematological cancer could prove useful for ruling out invasive aspergillosis. In contrast, the PPV of the assay was low. An explanation for this finding is that MycAssay™ Aspergillus is able to detect *Aspergillus* DNA in samples from colonized patients or from patients with other forms of aspergillosis, including chronic and allergic pulmonary aspergillosis [36], [37] (Table 5). Another explanation may be the low prevalence of invasive pulmonary aspergillosis found in the study population.

The main limitation of our study is that we were not able to include histologically proven cases because no lung biopsies were collected or post-mortem examinations performed. This is a common limitation in studies evaluating the role of new diagnostic tools for the diagnosis of invasive aspergillosis. Therefore, the patients were diagnosed with invasive aspergillosis based on microbiological or radiological data. The fact that *Aspergillus* was isolated from lower respiratory tract samples in all patients could explain the high correlation found between fungal culture and PCR. In our patients, MycAssay™ Aspergillus did not allow us to diagnose new cases that could be missed with fungal culture. Another limitation is that we classified our patients using the EORTC criteria, which were specifically developed for patients with cancer. EORTC criteria were chosen in the absence of specific criteria for non-cancer patients.

We conclude that MycAssay™ Aspergillus performed on lower respiratory tract samples showed high sensitivity for the diagnosis of invasive aspergillosis in patients without hematological cancer. Sensitivity increased when multiple samples were analyzed. Sensitivity was high for patients with COPD, which is an emerging risk population in some hospitals. MycAssay™ Aspergillus proved to be particularly useful for ruling out the diagnosis of invasive aspergillosis and significantly reduced time to diagnosis compared with conventional fungal culture. MycAssay™ Aspergillus should be used simultaneously with fungal culture of lower respiratory tract samples in order to provide additional data on antifungal susceptibility and ensure accurate identification of the isolates.
